# Estimates of Pandemic Influenza Vaccine Effectiveness in Europe, 2009–2010: Results of Influenza Monitoring Vaccine Effectiveness in Europe (I-MOVE) Multicentre Case-Control Study

**DOI:** 10.1371/journal.pmed.1000388

**Published:** 2011-01-11

**Authors:** Marta Valenciano, Esther Kissling, Jean-Marie Cohen, Beatrix Oroszi, Anne-Sophie Barret, Caterina Rizzo, Baltazar Nunes, Daniela Pitigoi, Amparro Larrauri Cámara, Anne Mosnier, Judith K. Horvath, Joan O'Donnell, Antonino Bella, Raquel Guiomar, Emilia Lupulescu, Camelia Savulescu, Bruno C. Ciancio, Piotr Kramarz, Alain Moren

**Affiliations:** 1 EpiConcept, Paris, France; 2 Réseau des GROG/Open Rome, Paris, France; 3 National Center for Epidemiology, Budapest, Hungary; 4 Health Protection Surveillance Centre, Dublin, Ireland; 5 European Programme for Intervention Epidemiology Training (EPIET), European Centre for Disease Prevention and Control, Stockholm, Sweden; 6 National Centre for Epidemiology, Surveillance and Health Promotion, Istituto Superiore di Sanità, Roma, Italy; 7 Instituto Nacional de Saúde Dr Ricardo Jorge, Lisbon, Portugal; 8 Cantacuzino Institute, National Institute of Research – Development for Microbiology and Immunology, Bucharest, Romania; 9 Universitatea de Medicina si Farmacie Carol Davila, Bucharest, Romania; 10 National Centre for Epidemiology, Instituto de Salud Carlos III, Madrid, Spain; 11 European Centre for Disease Prevention and Control (ECDC), Stockholm, Sweden; George Washington University, United States of America

## Abstract

**Background:**

A multicentre case-control study based on sentinel practitioner surveillance networks from seven European countries was undertaken to estimate the effectiveness of 2009–2010 pandemic and seasonal influenza vaccines against medically attended influenza-like illness (ILI) laboratory-confirmed as pandemic influenza A (H1N1) (pH1N1).

**Methods and Findings:**

Sentinel practitioners swabbed ILI patients using systematic sampling. We included in the study patients meeting the European ILI case definition with onset of symptoms >14 days after the start of national pandemic vaccination campaigns. We compared pH1N1 cases to influenza laboratory-negative controls. A valid vaccination corresponded to >14 days between receiving a dose of vaccine and symptom onset. We estimated pooled vaccine effectiveness (VE) as 1 minus the odds ratio with the study site as a fixed effect. Using logistic regression, we adjusted VE for potential confounding factors (age group, sex, month of onset, chronic diseases and related hospitalizations, smoking history, seasonal influenza vaccinations, practitioner visits in previous year). We conducted a complete case analysis excluding individuals with missing values and a multiple multivariate imputation to estimate missing values. The multivariate imputation (*n* = 2902) adjusted pandemic VE (PIVE) estimates were 71.9% (95% confidence interval [CI] 45.6–85.5) overall; 78.4% (95% CI 54.4–89.8) in patients <65 years; and 72.9% (95% CI 39.8–87.8) in individuals without chronic disease. The complete case (*n* = 1,502) adjusted PIVE were 66.0% (95% CI 23.9–84.8), 71.3% (95% CI 29.1–88.4), and 70.2% (95% CI 19.4–89.0), respectively. The adjusted PIVE was 66.0% (95% CI −69.9 to 93.2) if vaccinated 8–14 days before ILI onset. The adjusted 2009–2010 seasonal influenza VE was 9.9% (95% CI −65.2 to 50.9).

**Conclusions:**

Our results suggest good protection of the pandemic monovalent vaccine against medically attended pH1N1 and no effect of the 2009–2010 seasonal influenza vaccine. However, the late availability of the pandemic vaccine and subsequent limited coverage with this vaccine hampered our ability to study vaccine benefits during the outbreak period. Future studies should include estimation of the effectiveness of the new trivalent vaccine in the upcoming 2010–2011 season, when vaccination will occur before the influenza season starts.

*Please see later in the article for the Editors' Summary*

## Abstract 2

Results from a European multicentre case-control study reported by Marta Valenciano and colleagues suggest good protection by the pandemic monovalent H1N1 vaccine against pH1N1 and no effect of the 2009–2010 seasonal influenza vaccine on H1N1.

## Editors' Summary

**Background:**

Following the World Health Organization's declaration of pandemic phase six in June 2009, manufacturers developed vaccines against pandemic influenza A 2009 (pH1N1). On the basis of the scientific opinion of the European Medicines Agency, the European Commission initially granted marketing authorization to three pandemic vaccines for use in European countries. During the autumn of 2009, most European countries included the 2009–2010 seasonal influenza vaccine and the pandemic vaccine in their influenza vaccination programs.

The Influenza Monitoring Vaccine Effectiveness in Europe network (established to monitor seasonal and pandemic influenza vaccine effectiveness) conducted seven case-control and three cohort studies in seven European countries in 2009–2010 to estimate the effectiveness of the pandemic and seasonal vaccines. Data from the seven pilot case-control studies were pooled to provide overall adjusted estimates of vaccine effectiveness.

**Why Was This Study Done?:**

After seasonal and pandemic vaccines are made available to populations, it is necessary to estimate the effectiveness of the vaccines at the population level during every influenza season. Therefore, this study was conducted in European countries to estimate the pandemic influenza vaccine effectiveness and seasonal influenza vaccine effectiveness against people presenting to their doctor with influenza-like illness who were confirmed (by laboratory tests) to be infected with pH1N1.

**What Did the Researchers Do and Find?:**

The researchers conducted a multicenter case-control study on the basis of practitioner surveillance networks from seven countries—France, Hungary, Ireland, Italy, Romania, Portugal, and Spain. Patients consulting a participating practitioner for influenza-like illness had a nasal or throat swab taken within 8 days of symptom onset. Cases were swabbed patients who tested positive for pH1N1. Patients presenting with influenza-like illness whose swab tested negative for any influenza virus were controls.

Individuals were considered vaccinated if they had received a dose of the vaccine more than 14 days before the date of onset of influenza-like illness and unvaccinated if they were not vaccinated at all, or if the vaccine was given less than 15 days before the onset of symptoms. The researchers analyzed pandemic influenza vaccination effectiveness in those vaccinated less than 8 days, those vaccinated between and including 8 and 14 days, and those vaccinated more than 14 days before onset of symptoms compared to those who had never been vaccinated.

The researchers used modeling (taking account of all potential confounding factors) to estimate adjusted vaccine effectiveness and stratified the adjusted pandemic influenza vaccine effectiveness and the adjusted seasonal influenza vaccine effectiveness in three age groups (<15, 15–64, and ≥65 years of age).

The adjusted results suggest that the 2009–2010 seasonal influenza vaccine did not protect against pH1N1 illness. However, one dose of the pandemic vaccines used in the participating countries conferred good protection (65.5%–100% according to various stratifications performed) against pH1N1 in people who attended their practitioner with influenza-like illness, especially in people aged <65 years and in those without any chronic disease. Furthermore, good pandemic influenza vaccine effectiveness was observed as early as 8 days after vaccination.

**What Do These Findings Mean?:**

The results of this study provide early estimates of the pandemic influenza vaccine effectiveness suggesting that the monovalent pandemic vaccines have been effective. The findings also give an indication of the vaccine effectiveness for the Influenza A (H1N1) 2009 strain included in the 2010–2011 seasonal vaccines, although specific vaccine effectiveness studies will have to be conducted to verify if similar good effectiveness are observed with 2010–2011 trivalent vaccines. However, the results of this study should be interpreted with caution because of limitations in the pandemic context (late timing of the studies, low incidence, low vaccine coverage leading to imprecise estimates) and potential biases due the study design, confounding factors, and missing values. The researchers recommend that in future season studies, the sample size per country should be enlarged in order to allow for precise pooled and stratified analyses.

**Additional Information:**

Please access these websites via the online version of this summary at http://dx.doi.org/10.1371/journal.pmed.1000388.

- The World Health Organization has information on H1N1 vaccination
- The US Centers for Disease Control and Prevention provides a fact sheet on the 2009 H1N1 influenza virus
- The US Department of Health and Human services has a comprehensive website on flu
- The European Centre for Disease Prevention and Control provides information on 2009 H1N1 pandemic
- The European Centre for Disease Prevention and Control presents a summary of the 2009 H1N1 pandemic in Europe and elsewhere

## Introduction

Following the World Health Organization's declaration of pandemic phase six in June 2009, manufacturers developed vaccines against pandemic influenza A (H1N1) 2009 (pH1N1). On the basis of advice from the European Medicine Agency (EMA), the European Commission initially granted marketing authorization for three pandemic vaccines to be used in European Union (EU) countries. In selected countries including France, Hungary, and Romania, national regulatory authorities provided a licence for additional vaccines. Early clinical trials showed that the pandemic vaccines elicited good immunological responses after the first dose [1]–[3]. However, as strong immunogenicity does not always result in robust vaccine effectiveness (VE), it was important to estimate the effectiveness of the vaccine at the population level.

In the first months of the pandemic, various studies assessed the effect of the 2008–2009 seasonal influenza vaccine on pH1N1 related outcomes. The results were controversial: a hospital-based case-control study in Mexico suggested a protective effect of the vaccine against pH1N1 hospitalization [4], while studies in Australia and the United States did not find any effect of 2008–2009 seasonal influenza vaccine on the risk of medically attended pH1N1 illness [5],[6]. Studies in Canada suggested an increased risk of pandemic H1N1 infection following receipt of the seasonal influenza vaccine [7].

During the autumn of 2009, most EU member states included the 2009–2010 seasonal influenza vaccine and the pandemic H1N1 influenza vaccine in their influenza vaccination programmes. The groups targeted by the seasonal and pandemic vaccination programmes differed among member states. In some risk groups, both seasonal and pandemic vaccines were recommended.

The Influenza Monitoring Vaccine Effectiveness in Europe (I-MOVE) network was established with the aim of monitoring seasonal and pandemic influenza vaccine effectiveness (PIVE) [8]. During the 2008–2009 pilot season, five case-control and two cohort studies were conducted in six EU member states to estimate the VE of the 2008–2009 seasonal vaccine [9],[10]. Data from the five pilot case-control studies were pooled to provide an overall adjusted VE [11].

In 2010, to estimate the PIVE against medically attended influenza-like illness (ILI) laboratory confirmed as p1H1N1, we undertook a multicentre case-control study based on sentinel practitioner surveillance networks from seven study sites (France, Hungary, Ireland, Italy, Romania, Portugal, and Spain). A secondary objective of the study was to estimate the effectiveness of the 2009–2010 seasonal influenza vaccine against medically attended ILI laboratory confirmed as pH1N1.

## Methods

The study was conducted within the context of the existing European Influenza Surveillance Network (EISN) [12]. At the seven study sites, EISN sentinel primary care practitioners were invited to participate in the study. In Portugal and Italy, practitioners other than those participating in EISN, were also invited to participate.

The study population consisted of patients consulting a participating practitioner for ILI (six sites) or acute respiratory infection (ARI) (France) and having a nasal or throat swab taken within an interval of less than 8 d after symptom onset. In Hungary, the study population was restricted to patients aged more than 17 y. In Italy, the study population was restricted to patients who belonged to the groups for which the pandemic vaccine was recommended.

In five of the seven study sites practitioners used a systematic random sample to select the patients to swab. In Ireland each participating practice was asked to take a nasal or throat swab from five patients presenting with ILI each week. In France, each practitioner had an age group assigned and swabbed the first ARI patient of the week in the allocated age group.

A case of pandemic influenza A (H1N1) 2009 (pH1N1 case) was an ILI patient (defined according to the EU case definition as sudden onset of symptoms and at least one of the following four systemic symptoms: fever or feverishness, malaise, headache, myalgia, and at least one of the following three respiratory symptoms: cough, sore throat, shortness of breath) [13] who was swabbed and tested positive for the pH1N1 using real-time (RT) PCR or culture. Controls were ILI patients who were swabbed and tested negative for any influenza virus.

Swabs were tested for influenza at the respective countries' National Influenza Reference Laboratory. In France, Italy, and Spain, tests were also conducted in other laboratories participating in the National Influenza Sentinel Surveillance System.

For pandemic and seasonal influenza vaccine, individuals were considered vaccinated if they had received a dose of the vaccine more than 14 d before the date of onset of ILI symptoms and unvaccinated if they had received no vaccine or the vaccine was given less than 15 d before the onset of ILI symptoms. For pandemic vaccination we also estimated the PIVE among those vaccinated less than 8 d, those vaccinated between (and including) 8 and 14 d, and those vaccinated more than 14 d before onset of symptoms compared to those never vaccinated. Participating sentinel practitioners conducted face-to-face interviews with ILI patients using pilot-tested country-specific standardised questionnaires. The variables collected included ILI signs and symptoms, date of onset of symptoms, pandemic and 2009–2010 seasonal vaccination status including date of vaccination, and a list of potential confounding factors: age, sex, presence of chronic condition(s), pregnancy, obesity (not collected in France), severity of chronic disease using the number of hospitalizations for the chronic disease(s) in the previous 12 mo as a proxy, smoking history (nonsmoker, past, current smoker), number of practitioner visits in the previous 12 mo, influenza antiviral use before swabbing, and seasonal influenza vaccination in the previous two seasons. Vaccination status was ascertained using the practitioners' medical records or during the patient interview.

Each of the seven study teams entered and validated data. Validation of the vaccination status and of other variables was attempted by contacting the practitioner or by checking existing vaccination registries in the case of missing information.

The study teams sent anonymised databases of ILI cases recruited to the EpiConcept coordination team. The coordination team checked the data for inconsistencies, outliers, and logical errors and created a common dataset restricted to individuals meeting the EU ILI case definition, with onset of ILI symptoms more than 14 d after the start of the pandemic vaccination in each country. For each study site, we included in the common dataset records up to the week that preceded two consecutive weeks in which none of the recruited patients tested positive for pH1N1. We excluded individuals who tested positive for influenza A but had a nontypeable strain, those testing positive for other strains of influenza A or for influenza B, and those with missing information on laboratory results.

We compared the characteristics of cases and controls using the Fisher exact test or Mann-Whitney test as appropriate. We estimated the pooled seasonal influenza vaccine effectiveness (SIVE) and PIVE as 1 minus the odds ratio (OR) using a one-stage method with the study site as fixed effect in the model. To estimate adjusted VE, we used logistic regression models including all potential confounding factors.

We first conducted the analysis excluding all individuals with missing values (complete case analysis). We then estimated missing data for pandemic vaccination status and covariates using the multiple multivariate imputation by chained equations procedure in Stata [14]. We used missing at random assumptions. We used all predictors together to impute the missing values and independently analysed 20 copies of the data using 30 cycles of regression.

We stratified the adjusted PIVE and SIVE according to three age groups (<15, 15–64, and ≥65 y of age) and the adjusted PIVE by presence of chronic disease. We split the study period into two periods (early and late phase) using the date of symptom onset of the median case in each of the study sites and estimated PIVE for each of the phases.

We conducted all statistical analysis using Stata version 10.1 (StataCorp LP).

According to country-specific requirements for ethical approval, all participants provided oral or written consent.

## Results

In the seven participating countries, influenza activity peaks were reached between week 43 (Ireland) and week 50 (Hungary, Romania) (Figure S1) of 2009. Of the six vaccines used at the seven study sites, three were adjuvanted (Table S1). The first country to start a pandemic vaccination campaign was Hungary (week 40) and the last was Romania (week 48) (Figure S1; Table 1).

**Table 1:** Timing of key events in the 2009–2010 influenza season relevant to the I-MOVE study.

Country	Week of Maximum Incidence of ARI^a^ or ILI^b^	Week of Start of Pandemic Vaccination Campaign	Week of Inclusion of First ILI Case in the Study	Date of Inclusion of First ILI Case in the Study
**France**	49^a^	43	45	04/11/2009
**Hungary**	50^b^	40	50	09/12/2009
**Ireland**	43^b^	45	47	17/11/2009
**Italy**	46^b^	43	47	17/11/2009
**Portugal**	46^b^	44	46	12/11/2009
**Romania**	50^a^	48	53	03/01/2010
**Spain**	46^b^	46	48	01/12/2009

a Sentinel systems reporting ARIs.

b Sentinel systems reporting ILI.

A total of 1,114 practitioners agreed to participate in the study. Within the study period, 699 of the practitioners recruited 2,926 patients who met the EU ILI case definition and who were swabbed less than 8 d after symptom onset (Table 2). After excluding 17 individuals with non-subtypeable influenza A, one positive for influenza B, and six with missing information on laboratory results, a total of 2,902 ILI patients were included in the analysis (Figure S2). Among these patients, 918 (31.6%) were positive for pH1N1 (ranging from 15.2% in Hungary to 38.1% in France).

**Table 2:** Practitioners and patient recruitment in the 2009–2010 influenza season relevant to the I-MOVE study.

Study Site	Practitioners in the National Sentinel System, *n*	Practitioners Accepting to Participate in the Study, *n*	Practitioners Recruiting at Least One ILI Patient^a^, *n*	ILI Patients^a^ Recruited by Practitioners, *n*	Inclusion Period for the Study^b^	ILI Patients Included in the Study Positive for Influenza A (H1N1) 2009^c^, *n*		ILI Patients Included in the Study Negative for Influenza A (H1N1) 2009^c^, *n*	
						Total	Vaccinated	Total	Vaccinated
**France**	550	550	429	1,908	4/11/2009–28/2/2010	720	3	1,172	63
**Hungary**	168	87	63	361	8/12/2009–14/3/2010	55	6	306	100
**Ireland**	137	48	19	77	17/11/2009–10/1/2010	29	1	48	2
**Italy**	1,163	47	21	69	03/11/2009–13/12/2009	18	0	44	0
**Portugal**	150	53	32	186	10/11/2009–21/2/2010	31	0	155	10
**Romania**	270	102	12	24	17/12/2009–31/1/2010	5	1	19	1
**Spain**	880	227	123	301	01/12/2009–7/2/2010	60	1	240	9
**Total**	3,318	1,114	699	2,926		918	12	1,984	185

a ILI patients meeting the EU case definition, swabbed <8 d after onset of symptoms within the study period.

b For each study site, from 15 d after the start of the vaccination campaign up to the week that preceded 2 consecutive weeks in which none of the ILI patients recruited tested positive for influenza A (H1N1) 2009 recruited. In Hungary, the start of the study period was the week of receiving the agreement from the Ethics Committee.

c ILI patients in the study after excluding those having tested previously to pH1N1, those positive to other influenza virus, and those with missing information on laboratory results.

197 individuals (6.9%) had received at least one dose of pandemic vaccine more than 14 d before the date of symptom onset (ranging from 0.0% in Italy to 29.4% in Hungary). 11 of them had received two doses. Out of the 197 individuals vaccinated, vaccine brand was documented for 195. Among them, 155 (79.5%) had received an adjuvanted vaccine and 40 (20.5%) a nonadjuvanted vaccine.

The median age was lower in cases (12 y) than in controls (27 y). The delay between onset of symptoms and swabbing was shorter in cases than in controls (Table 3). The proportion of individuals presenting with fever, headache, or cough was higher among cases than among controls. Compared to cases, a higher proportion of controls had diabetes, heart disease, and were hospitalised at least once for their chronic disease in the previous 12 mo. A higher proportion of controls were current or past smokers, vaccinated with the 2009–2010 seasonal influenza vaccine, and vaccinated against influenza in the previous 2 y. The median number of practitioner visits in the previous 12 mo was three for cases (ranging from 0 to 22) and four for controls (ranging from 0 to 44) (Table 3). A total of 12 pH1N1 cases were vaccinated with the pandemic vaccine more than 14 d before symptom onset. Two of these cases were under 15 y of age, three were 65 y of age or older, and the remaining seven were aged 15 to 64 y. None of the cases had received two doses of the pandemic vaccine. In two of the seven studies there were no vaccinated individuals among the recruited cases (Table 2).

**Table 3:** Pandemic influenza A (H1N1) 2009 cases and test-negative controls included in the study by patient characteristics.

Patient Characteristics	Cases *n* = 918	Test-Negative Controls *n* = 1,984	*p*-Value
**Median age, y**	12	27	<0.001^a^
**Age group, y, ** ***n*** **/total ** ***n*** ** (%)**			
0–4	180/917 (19.6)	520/1,978 (26.3)	<0.001^b^
5–14	326/917 (35.6)	195/1,978 (9.9)	
15–64	393/917 (42.9)	1,069/1,978 (54.0)	
≥65	18/917 (2.0)	194/1,978 (9.8)	
**Female sex, ** ***n*** **/total ** ***n*** ** (%)**	485/912 (53.2)	1,005/1,971 (51.0)	0.279^b^
**Symptoms, ** ***n*** **/total ** ***n*** ** (%)**			
Fever	903/918 (98.4)	1,842/1,957 (94.1)	<0.001^b^
Headache	611/907 (67.4)	1,194/1,936 (61.7)	0.003
Cough	869/914 (95.1)	1,718/1,964 (87.5)	<0.001^b^
Sore throat	539/914 (59.0)	1,340/1,945 (68.9)	<0.001^b^
**Days between onset of symptoms and swabbing, ** ***n*** **/total ** ***n*** ** (%)**			
0	111/918 (12.1)	212/1,984 (10.7)	<0.001^b^
1	512/918 (55.8)	987/1,984 (49.7)	
2	201/918 (21.9)	454/1,984 (22.9)	
3	70/918 (7.6)	181/1,984 (9.1)	
4	17/918 (1.9)	77/1,984 (3.9)	
5	6/918 (0.7)	36/1,984 (1.8)	
6	1/918 (0.1)	21/1,984 (1.1)	
7	0/918 (0.0)	16/1,984 (0.8)	
**Diabetes, ** ***n*** **/total ** ***n*** ** (%)**	8/690 (1.2)	72/1,670 (4.3)	<0.001^b^
**Heart disease, ***n***/total ***n*** (%)**	20/688 (2.9)	198/1,670 (11.9)	<0.001^b^
**Any hospitalization in the previous 12 mo for chronic diseases, ** ***n*** **/total ** ***n*** ** (%)**	5/680 (0.7)	37/1,739 (2.3)	0.005^b^
**Smoker, ** ***n*** **/total ** ***n*** ** (%)**			
Current	35/814 (4.3)	176/1,739 (10.1)	<0.001^b^
Former	80/814 (9.8)	244/1,739 (14.0)	
Never	699/814 (85.9)	1319/1,739 (75.8)	
**Pandemic vaccination, ** ***n*** **/total ** ***n*** ** (%)**	12/895 (1.3)	185/1,940 (9.5)	<0.001^b^
**Seasonal vaccination, 2009–2010, ** ***n*** **/total ** ***n*** ** (%)**	56/913 (6.1)	240/1,975 (12.2)	<0.001^b^
**Any influenza vaccination in the previous two seasons, ** ***n*** **/total ** ***n*** ** (%)**	56/516 (10.9)	213/1,316 (16.2)	0.003^b^
**Median number of GP visits in the previous 12 mo**	3	4	<0.001^a^

a Nonparametric test of the median.

b Two-sided Fisher exact test.

### Pandemic Vaccine Effectiveness

We included 1,502 individuals in the pooled complete case analysis. The overall PIVE adjusted for all potential confounding factors was 66.0%, 71.3% in those aged <65 y, and 70.2% in those with no chronic disease (Table 4).

**Table 4:** Pooled crude and adjusted PIVE.

Complete Case and Imputed Data Analysis	Crude and Adjusted PIVE Estimates	Included Population	*n*	Percent PIVE	95% CI
**Complete case analysis** a	Crude^b^	All	1,502	79.0	55.8–90.0
		<65 y	1,367	83.3	61.2–92.8
		15–64 y	912	76.6	44.7–90.1
		<15 y	455	100	58.2–100.0^c^
		No chronic disease	1,190	81.5	53.0–92.7
	Adjusted model^d^	All	1502	66.0	23.9–84.8
		<65 y	1,367	71.3	29.1–88.4
		15–64 y	912	65.5	12.3–86.5
		<15 y	455	100	Not calculable^e^
		No chronic disease	1,190	70.2	19.4–89.0
**Imputed data** f	Crude^b^	All	2,902	82.8	68.6–90.6
		<65 y	2,688	86.9	73.9–93.4
		15–64 y	1,463	80.6	57.2–91.2
		<15 y	1,218	94.2	75.6–98.6
		No chronic disease	2,354	84.6	67.7–92.7
	Adjusted model^d^	All	2,902	71.9	45.5–85.5
		<65 y	2,688	78.4	54.3–89.8
		15–64 y	1,463	73.3	36.7–88.7
		<15y	1,218	84.8	31.0–96.6
		No chronic disease	2,354	72.9	39.7–87.8

a Excluding individuals with missing values.

b Study site included in the model as fixed effect.

c Exact logistic regression estimates with zero cases vaccinated.

d Model adjusted for 2009–2010 seasonal influenza vaccination, any influenza vaccination in previous two seasons, presence of at least one chronic disease, sex, at least one hospitalization for chronic disease in the previous 12 mo, current smoker, age group, practitioner visits in previous 12 mo (0, 1–4, and 5+ visits), month of symptom onset (note: in the 15–64 y stratum no adjustment for age group; in the “no chronic disease” stratum no adjustment for chronic disease or hospitalizations for chronic disease).

e If one of the cases would have been vaccinated, the estimated PIVE would be 85.2% (95% CI 30.0–98.3).

f Missing data imputed using imputation using chained equations.

In the pooled analysis with imputed data, we included all 2,902 individuals. The overall PIVE adjusted for all potential confounding factors was 71.9%, 78.4% in those aged <65 y, and 72.9% in those with no chronic diseases (Table 4). PIVE was 79.3% (95% confidence interval [CI] 4.7–95.9) in the early phase and 68.8% (95% CI 35.8–84.8) in the late phase of the study.

We analysed an intermediate dataset that included 2,073 records after removing those with missing values in the variables that changed the odds ratio of being vaccinated by more than 5% in the complete case or multiple imputation analysis (age group, number of practitioner visits in the previous 12 mo, 2009-2010 seasonal influenza vaccine, and month of symptom onset). The PIVE adjusted for these variables was 72.4% (95% CI 44.1–86.4) overall, 80.1% (95% CI 54.8–91.2) in those aged <65 y, and 73.4% (95% CI 35.6–89.0) in those with no chronic disease.

Using 30 d as the cut off to start the inclusion of ILI patients in the study instead of 15 d after the start of the vaccination campaigns did not change the PIVE estimates (Table S6). In the complete case analysis, taking into account different delays between date of vaccination and date of onset of ILI symptoms, the overall PIVE was 66.0% for 8–14 d and 66.9% for more than 14 d (Table 5).

**Table 5:** Pooled crude and adjusted PIVE, according to categories based on delay between date of vaccination and date of onset of symptoms.

Crude and Adjusted PIVE Estimates	Included Population	Definition of Delay Vaccination—Onset of ILI Symptoms	*n*	Percent PIVE	95% CI
**Crude** a	All	<8 d	1,502	20.6	−157.9 to 75.5
		8–14 d		59.8	−85.3 to 91.3
		>14 d		79.2	56.3 to 90.1
	<65 y	<8 d	1,367	15.7	−18.1 to 74.7
		8–14 d		57.6	−97.6 to 90.9
		>14 d		83.5	61.5 to 92.9
**Adjusted model** b	All	<8 d	1,502	18.8	−183.4 to 76.7
		8–14 d		66.0	−69.9 to 93.2
		>14 d		66.9	26.0 to 85.2
	<65 y	<8 d	1,367	15.5	−198.1 to 76.1
		8–14 d		66.6	−70.8 to 93.5
		>14 d		72.0	30.8 to 88.7

a Study site included in the model as fixed effect.

b Model adjusted for 2009–2010 seasonal influenza vaccine, any influenza vaccination in previous two seasons, presence of at least one chronic disease, sex, at least one hospitalization for chronic disease in the previous 12 mo, current smoker, age group, practitioner visits in previous 12 mo (0, 1–4, and 5+ visits), month of symptom onset.

### Vaccine Effectiveness of the 2009–2010 Seasonal Vaccine

A total of 296 individuals (10.2%) had received the 2009–2010 seasonal vaccine more than 14 d before the date of symptom onset (Table 3). The SIVE estimates adjusted for all potential confounding factors was 9.9% in the complete-case analysis and −1.5% in the multiple imputation analysis (Table 6).

**Table 6:** Pooled crude and adjusted 2009–2010 seasonal VE, multicentre case-control study, influenza season 2009–2010, seven European Union study sites.

Complete Case and Imputed Data Analysis	Crude and Adjusted PIVE Estimates	Included Population	*n*	Percent VE	95% CI
**Complete case analysis** a	Crude^b^	All	1,502	47.5	21.3 to 65.0
		<65 y	1,367	47.0	14.0 to 67.4
	Adjusted model^c^	All	1,502	9.9	−65.2 to 50.9
		<65 y	1,367	31.4	−34.4 to 65.0
**Imputed data** d	Crude^b^	All	2,902	40.6	18.6 to 56.7
		<65 y	2,688	25.6	−7.3 to 48.4
	Adjusted model^c^	All	2,902	−1.5	−67.0 to 38.3
		<65 y	2,688	9.8	−57.2 to 48.3

a Excluding individuals with missing values.

b Study site included in the model as fixed effect.

c Model adjusted for 2009–2010 pandemic influenza vaccination, any influenza vaccination in previous two seasons, presence of at least one chronic disease, sex, at list one hospitalization for chronic disease in the previous 12 mo, current smoker, age group, practitioner visits in previous 12 mo (0, 1–4, and 5+ visits), month of symptoms onset.

d Missing data imputed using imputation using chained equations.

## Discussion

Using sentinel practitioner networks in seven EU countries, we estimated the effectiveness of the 2009–2010 pandemic and seasonal influenza vaccines. The pooled results suggest that one dose of a pandemic vaccine conferred good protection against medically attended pH1N1 ILI (65.5%–100% according to the various stratified analyses performed). The PIVE was higher in persons aged <65 y old and in those without any chronic disease. Furthermore, the PIVE point estimates suggest a good PIVE as early as 8 d after vaccination. During the study period, the 2009–2010 seasonal vaccine seems to have had no effect on pH1N1 illness.

We believe these results should be interpreted with caution for reasons including the late timing of the studies relative to pandemic vaccine rollout, low incidence of medically attended H1N1 illness, low vaccine coverage and potential biases due to the test-negative design, confounding factors, and missing values.

### Pandemic Context

One of the major limitations of the study is the timing of vaccination during the pandemic. In most participating countries, the pandemic vaccination campaigns and therefore the recruitment in the study sites started during the pandemic or after the peak of the pandemic.

As a consequence, part of the population had acquired natural immunity to the pandemic H1N1 influenza strain before the start of the studies. If this natural immunity differed between those who were later vaccinated and those who were not, this could have biased the PIVE. In particular, if vaccinated persons had a higher risk of infection before vaccination (e.g., children), we might have overestimated the PIVE. We may not have totally controlled for this indication bias by adjusting for age and time of recruitment. Only a cohort study design including a sero-prevalence component at the start of the study can help in quantifying this bias, which is likely to affect all studies conducted during the pandemic.

Within each country, eligible groups were not offered vaccination at the same time (Table S2). We could not restrict our analysis to the time at which individuals became eligible for vaccination as most sites did not include the necessary information to identify them. We may therefore have included individuals for whom vaccination was not or not yet indicated. Consequently, we may have inflated the number of cases unvaccinated in the early phase of the study and overestimated the PIVE. The potential biases introduced by including them cannot be quantified in our study and should be measured with cohort studies using large databases. However, the simulations we carried out suggest that such a bias may be minimal in a situation with low incidence and low vaccine coverage (unpublished data).

The low incidence of medically attended H1N1 influenza infection and the low pandemic influenza vaccination coverage in all study sites (Table S3) led to a small number of vaccinated cases and limited the statistical power of each of the stratified analyses. The low vaccination coverage did not allow PIVE estimation by vaccine brand. We computed PIVE by target groups for vaccination (age groups, chronic diseases). All estimates were above 60% but had very large CIs.

We could only estimate the effect of one dose of the pandemic vaccine because of the small number of individuals who had received two doses.

In countries where adjuvanted and nonadjuvanted vaccines were used, each vaccine was recommended for a different target group and marketed at different times (Tables S1 and S2). It was not possible to identify different target groups which precluded estimating effectiveness according to the vaccine type.

Our estimates of the 2009–2010 pandemic vaccine apply only to the study period, which is 15 d after the start of the pandemic vaccination campaigns. Using 30 d after the start of the pandemic vaccination campaigns as the study period, did not change the estimates (Table S6).

### Study Design

Our results are based on data from seven European countries sharing the same protocol and definition of variables. The pooled data resulted in a sample size with enough power to provide precise overall crude and adjusted pooled estimates.

### Misclassification

We observed shorter delays between onset of symptom and swabbing in cases than in controls. As the probability of influenza detection decreases with time since onset [15], we may have misclassified as controls some influenza cases who tested negative. If vaccinated cases develop milder illness and seek medical help later, the vaccination coverage in the control group will be inflated resulting in a higher PIVE. Similarly, if unvaccinated cases tend to consult their general practitioner (GP) later because of their health-seeking behaviour, the PIVE will be underestimated. On the other hand, because cases are less likely to be vaccinated, the vaccine coverage among controls will decrease by having cases misclassified as controls and the PIVE will be underestimated. Restricting the analysis to ILI patients tested within 4 d of onset of symptoms, PIVE estimates did not change (Table S4). In addition, in our studies, 91% of the ILI patients were swabbed less than 4 d after onset of ILI symptoms.

### Information

In the complete case analysis, we excluded 1,400 individuals with no information for at least one of the variables (losing one-third of vaccinated cases).

In the 2,902 observations, the highest proportion of missing values were for number of visits to a GP in the past 12 mo (27.5%) and influenza vaccination in the past two seasons (36.9%) (Table S7). Missing values for certain covariates were associated with the outcome and with pandemic influenza vaccination (unpublished data) and therefore we could not assume that our missing values fell into the category of missing completely at random (MCAR) [14]. To reduce the potential bias we used a method of multiple imputation by chained equations procedure in which values are imputed according to associations observed between many other variables (including confounders) and the missing variable. We were able to use a large number of variables for the imputation, including key variables such as week of symptom onset, outcome, study site, and vaccination variables. We also conducted the analysis excluding the 67 individuals with no information about pandemic vaccination status and the results did not change (adjusted PIVE 72.9%, 95% CI 46.7–85.6).

The PIVE estimated using the complete case analysis was slightly lower than the estimates using the dataset with imputed data (absolute differences ranging from 3.6% to 7.6%). However, all PIVE point estimates were greater than 65%. Given the smaller sample size, the PIVE estimates from the complete case analysis are less precise.

We further checked the outcome of the imputation by comparing the imputed values for pandemic vaccination against a validation subset in France (more than 90% of the missing data coming from France). The proportion vaccinated in the validation set was similar to the imputed proportion of vaccinated (Chi^2^ test for differences in proportion: *p* = 0.749). Missing values remain a limitation in observational studies based on surveillance data. The use of randomly selected validation subsets with additional and verified information will help controlling for potential biases because of missing values.

The administration of both trivalent seasonal and monovalent pandemic vaccine may have made the ascertainment of vaccination status difficult. In six countries vaccination was mainly done by practitioners and we believe that they correctly documented vaccination status. In France, pandemic vaccinations were only done in pandemic vaccination centres where each individual received a vaccination card. Patients could remember if they had been to a pandemic vaccination centre and practitioners could in addition verify the vaccination cards.

### Selection

The test-negative design is a hybrid design approaching a density case-control study in which the effect measured would be an incidence density rate ratio [16]. The test-negative design differs from it since former influenza cases in the pandemic are not excluded from potential controls (ILI testing negative).

In studies using the test-negative design, GPs may be more likely to swab vaccinated ILI patients. In our studies the recruitment of an ILI patient was not left to the GP's decision. GPs from five out of the seven sites used systematic sampling to recruit and swab ILI patients. In Ireland practitioners were instructed to include five ILI patients per week without applying a systematic selection procedure. This could have introduced a selection bias if the inclusion criteria were linked to the vaccination status and to the case-control status. However, the participating practitioners in Ireland recruited fewer than five cases per week, suggesting that they recruited all patients consulting for ILI. In France, each practitioner recruited a specific age group for the study. Thus, ILI patients recruited may not have represented the age distribution of the ILI population consulting participating practitioners. This consideration could have biased the PIVE estimates if PIVE differed by age group. However, ILI cases recruited in the study by French participating practitioners have the exact same age distribution as all ILI cases consulting them (unpublished data). In addition, selection bias was further minimized since practitioners did not know the case or control status of the ILI patients at time of recruitment.

### Confounding

We limited the effect of potential confounding factors by adjusting for most of the confounding factors described in the literature. In all our estimates, the adjusted PIVE was lower than the crude PIVE (absolute differences ranging from 7.3% to 12.8%) suggesting some positive confounding. The main confounders identified were time and age groups (Table S5). Time was associated with vaccination status and outcome (lower vaccination coverage and higher influenza incidence at the beginning the study). During the influenza H1N1 pandemic, vaccination was a time-dependent variable and the vaccine coverage observed among controls increased over time. When splitting the study period, the adjusted PIVE for both early and late phases was above 68%. Further stratification of time was not possible due to small numbers (i.e., two cases vaccinated in the early phase and ten cases in the late phase).

In addition, the propensity to seek care and accept vaccination may have changed over time during the pandemic. We controlled for these potential changes by adjusting for month of onset of symptoms. Adjusting for week of onset did not change the PIVE estimates.

In the test-negative design the representativeness of the test-negative controls has not yet been validated [11],[17]–[20]. Some studies suggest that vaccine coverage among ILI testing negative is higher than in the community [10],[17]. However community does not represent the source population giving rise to cases because vaccination coverage varies with health-seeking behaviour. The test-negative design is believed to adjust for differences in health-seeking behaviour between cases and controls. To further control for this potential bias, we adjusted for the number of practitioner visits in the previous 12 mo. However this adjustment may not be appropriate if health-seeking behaviour differs between seasonal and pandemic influenza.

One of the symptoms included in the EU ILI case definition is the presence of sudden onset of symptoms: if vaccinated cases are less likely to have sudden onset of symptoms and consequently less likely to be recruited into the study than unvaccinated cases the PIVE would be overestimated.

Overall if there is still residual confounding due to the above factors we may still be overestimating PIVE.

### Pooled Analysis

Even though the seven study sites shared a similar protocol, we were unable to properly measure the heterogeneity between studies owing to the small sample size at study-site level. We could only use a one-stage pooling model that assumes that the effect (PIVE) is the same in all the studies [21]. Heterogeneity between studies may still exist as the result of the use of different vaccines, different target groups, and a potential different health-seeking behaviour. Therefore pooled estimates have to be interpreted with caution.

### Seasonal Vaccine

During the study period, the 2009–2010 seasonal vaccine seems to have had no effect on pandemic H1N1 influenza illness. The small number of ILI patients recruited in the ≥65 y age group and the small number of vaccinated patients among the <15-y-olds precluded making robust VE estimates in these age groups. In our study crude SIVE estimates are higher than adjusted SIVE estimates. Methods suggested for controlling such positive confounding include identifying an adjusted model leading to 0% VE before circulation of the virus and applying it to the seasonal peak [22]. Those models are not applicable to laboratory-confirmed outcomes.

Due to the controversial results of the effect of the 2008–2009 seasonal vaccine on pandemic H1N1-related outcomes [6],[7],[19], it would have been interesting to estimate the VE of the 2009–2010 seasonal vaccine during the peak of the pandemic and before the introduction of the pandemic vaccine; this was not done. In addition, the small sample size in our study does not allow measurement of any interaction between seasonal and pandemic vaccines.

The good PIVE estimates we observed may be affected by the test-negative design and its potential for bias and by the timing of the studies in the late phase of the pandemic. As a consequence, we cannot exclude that the PIVE we observed is overestimated. Despite these limitations we believe that results from all seven study sites are consistent in terms of the low number of vaccine failures. The good PIVE found in the study corroborates the strong immunogenicity results observed in clinical studies [1]–[3], and the preliminary estimates of PIVE in Germany [23], Castellón (Spain) [24], and Scotland [25].

In the past, similar studies using the test-negative design for seasonal vaccines have documented estimates ranging from 34% to 92% in seasons of good vaccine matching [10],[11],[17]–[20],[26]. The pandemic estimates we observed in 2009–2010 fit in the upper quartile of that distribution. However such a comparison is complicated by potential differences in health-seeking behaviours, age groups, and timing of studies.

This is the second year we have pilot tested a multicentre study using the test-negative design. In future influenza seasons the sample size per country will be enlarged in order to allow for precise pooled and stratified analyses. In addition the use of validation subsets, in which we collect more accurate and additional information in a subsample of the ILI patients, will be promoted.

I-MOVE is a unique network in Europe that is able to measure seasonal and pandemic VE even in periods of high workload like the 2009–2010 pandemic influenza season. On the basis of the experience of the pilot phase in 2008–2009, and despite the low pandemic vaccination coverage in the participating countries, the results of the multicentre case-control study have provided early estimates of the PIVE suggesting that the monovalent pandemic vaccines have been effective. Our findings also provide an indication of the VE for the A (H1N1) 2009 strain included in the 2010–2011 seasonal vaccines. Specific VE studies will have to be conducted to verify if similar good effectiveness estimates are observed with the 2010–2011 trivalent vaccines.

## Supporting Information

Figure S1 ILI and ARIs and cases and controls recruited, by week and country, multicentre case-control study, influenza season 2009–2010, seven European Union study sites.
(0.15 MB XLS)

Figure S2 Flowchart of data exclusion for pooled analysis, I-MOVE multicentre case-control studies 2009–2010.
(0.07 MB PDF)

Table S1 Pandemic vaccines used by study site, multicentre case-control study, influenza season 2009–2010.
(0.04 MB DOC)

Table S2 Priority groups for pandemic vaccination and date of start of the pandemic vaccination campaign by country study site, multicentre case-control study, influenza season 2009–2010, seven European Union study sites.
(0.06 MB DOC)

Table S3 Estimated pandemic vaccination coverages by country study site, multicentre case-control study, influenza season 2009–2010, seven European Union study sites.
(0.03 MB DOC)

Table S4 Pooled crude and adjusted PIVE restricted to ILI patients swabbed <4 d after onset of ILI symptom, multicentre case-control study, influenza season 2009–2010, seven European Union study sites.
(0.05 MB DOC)

Table S5 Logistic regression model: Full model for complete case analysis.
(0.11 MB DOC)

Table S6 Analysis using start of study period more than 30 d after the start of the study site-specific vaccination campaign.
(0.04 MB DOC)

Table S7 Number and proportion of observations (*n* = 2,902) with missing values by variable, influenza season 2009–2010, seven European Union study sites.
(0.03 MB DOC)

## Abbreviations

ARI: acute respiratory infection
CI: confidence interval
EU: European Union
GP: general practitioner
ILI: influenza-like illness
PIVE: pandemic influenza vaccine effectiveness
SIVE: seasonal influenza vaccine effectiveness
VE: vaccine effectiveness
