# Supplementary material for: Estimates of Pandemic Influenza Vaccine Effectiveness in Europe, 2009–2010: Results of Influenza Monitoring Vaccine Effectiveness in Europe (I-MOVE) Multicentre Case-Control Study
Source: PLoS Med. 2011 Jan 11;8(1):e1000388. doi: 10.1371/journal.pmed.1000388 (PMC3019108; doi:10.1371/journal.pmed.1000388)
Supplement: Figure S2 — Flowchart of data exclusion for pooled analysis, I-MOVE multicentre case-control studies 2009–2010. (0.07 MB PDF) [file pmed.1000388.s002.pdf]

Figure S2 - Flowchart of data exclusion for pooled analysis, I-MOVE multi-centre case control studies 2009-10

Data received	France: 2988	Hungary: 411	Ireland: 165*	Italy: 172	Portugal: 244	Romania: 97	Spain: 1504
Data cut-off: first date of vaccination campaign >14 days	-257	-0	-46	-0	-5	-9	-1047
	FR: 2731	HU: 411	IR: 119	IT: 172	PT: 239	RO: 88	ES: 457
Subtracting persons not adhering to EU ILI case definition	-677	-20	-19	-29	-20	-4	-63
	FR: 2054	HU: 391	IR: 100	IT: 143	PT: 219	RO: 84	ES: 394
Subtracting persons with an interval >7 days between symptoms and swabbing	-4	-1	-1	-0	-2	-2	-4
	FR: 2050	HU: 390	IR: 99	IT: 143	PT: 217	RO: 82	ES: 390
Exclusion of controls after ISO week of last case (onset weeks)	-142	-29	-22	-74	-31	-58	-89
	FR: 1908	HU: 361	IR: 77	IT: 69	PT: 186	RO: 24	ES: 301
Missing virus or subtype or seasonal influenza	-16	-0	-0	-7	-0	-0	-1
	FR: 1892	HU: 361	IR: 77	IT: 62	PT: 186	RO: 24	ES: 300

* 1 record excluded for Ireland as they had previous A(H1N1)v
