# Supplementary material for: Estimates of Pandemic Influenza Vaccine Effectiveness in Europe, 2009–2010: Results of Influenza Monitoring Vaccine Effectiveness in Europe (I-MOVE) Multicentre Case-Control Study
Source: PLoS Med. 2011 Jan 11;8(1):e1000388. doi: 10.1371/journal.pmed.1000388 (PMC3019108; doi:10.1371/journal.pmed.1000388)
Supplement: Table S1 — Pandemic vaccines used by study site, multicentre case-control study, influenza season 2009–2010. (0.04 MB DOC) [file pmed.1000388.s003.doc]

**Table S1:** Pandemic vaccines used by study site, multicentre case-control study, influenza season 2009-10

| **Vaccines** | **Adjuvant** | **Date Marketing authorisation2009** | **Countries** | | | | | | | | |
| --- | --- | --- | --- | --- | --- | --- | --- | --- | --- | --- | --- |
|  |  |  | **France** | **Hungary** | **Ireland** | **Italy** | **Portugal** | **Romania** | | **Spain** |  |
| **Cevalpan (Baxter)** | None | 2 Oct | Fr |  | Ir |  |  |  |  | | |
| **Focetria (Novartis)** | MF59 | 25 Sept | Fr |  |  | It |  |  | Sp | | |
| **Pandemrix (GSK)** | ASO3 | 25 Sept | Fr |  | Ir |  | Pt |  | Sp | | |
| **Fluval P**  **(Omninvest)** | Aluminium phosphate | 28 Sept |  | Hu |  |  |  |  |  | | |
| **Panenza**  **(Sanofi Pasteur)** | None | 16 Nov | Fr |  |  |  |  |  | Sp | | |
| **Cantgrip**  **(Cantacuzino)** | None | 26 Nov |  |  |  |  |  | Ro |  | | |
