# Supplementary material for: Estimates of Pandemic Influenza Vaccine Effectiveness in Europe, 2009–2010: Results of Influenza Monitoring Vaccine Effectiveness in Europe (I-MOVE) Multicentre Case-Control Study
Source: PLoS Med. 2011 Jan 11;8(1):e1000388. doi: 10.1371/journal.pmed.1000388 (PMC3019108; doi:10.1371/journal.pmed.1000388)
Supplement: Table S2 — Priority groups for pandemic vaccination and date of start of the pandemic vaccination campaign by country study site, multicentre case-control study, influenza season 2009–2010, seven European Union study sites. (0.06 MB DOC) [file pmed.1000388.s004.doc]

Table S2: Priority groups for pandemic vaccination and date of start of the pandemic vaccination campaign by country study-site, multicentre case-control study, influenza season 2009-10, seven EU study sites

| **Country** | **Target group** | **Date of start** |
| --- | --- | --- |
| France | Hospital health care workers | 20/10/2009 |
|  | Primary care health care workers | 02/11/2009 |
|  | High-risk people for seasonal influenza (6 months - 64 years)  + People with morbid obesity (6 months - 64 years)  + Professionals working with children under 3 years  + Other care professionals  + Household members of high-risk children under 6 months | 12/11/2009 |
|  | + Pregnant women (2nd and 3rd trimester)  + Non high-risk children 6-23 months | 20/11/2009 |
|  | Secondary school children | 25/11/2010 |
|  | + Primary school children  + High-risk people for seasonal influenza > - 64 years | Early December |
|  | Rest of the population, beginning with the youngest | 15/12/2010 |
| Hungary | + People aged over 12 months with chronic underlying conditions that put them at risk for severe disease. Underlying conditions, considered to be risk factors:  Chronic lung diseases, including moderate or severe asthma  Severe obesity or those with impaired lung function due to neuromuscular diseases  ...Cardiovascular diseases, except for well-treated hypertension  Congenital or acquired immune deficiency (included HIV-positives, or those suffering from malignant tumor)  Chronic diseases of the liver or kidney  Chronic metabolic disorders, including diabetes mellitus  + Pregnant women  + Health Care Workers  + Institutionalised people  + People working in the central command and control structures, home security services and essential services, critical infrastructure | 29/09/2009 |
|  | + Healthy children from 12 months to 18 years of age attending to kindergarten or school  + People older than 18 living in a dormitory  + Workforce of the educational institutions  + Household contacts and caregivers of children younger than 12 months of age | 02/11/2009 |
| Ireland | + At-risk groups aged 6 months up to 65 years of age  + Pregnant women in the 2nd and 3rd trimester and up to 6 weeks post partum or in the1st trimester with an additional risk factor  + Immunosuppressed individuals and household contacts of individuals with immunosuppression  + Residents of disability units regardless of whether they are in one of the medically at risk groups  + Individuals with significant physical or intellectual disability (including neurodevelopment conditions) | 19/10/2009 |
|  | + Health care staff  + Children aged 6 months – 5 years | 09/11/2009 |
|  | + Household contacts of children aged less than 6 months  + Children aged 5 – 18 years  + Adults aged 65 years and over | 30/11/2009 |
|  | All other groups | 01/02/2010 |
| Italy | + Health care personnel, personnel connected with essential services,  + Persons ≤65 with underlying conditions at high risk of severe or fatal complications due to influenza  + Pregnant women,  + Healthy children and adolescents aged between 2–18 years | Variable across regions, starting on 12/10/2009 |
|  | + Healthy adults  + Elderly with chronic diseases | 7/12/2009 |
| Portugal | Essential services, pregnant women individuals with chronic diseases 6 months – 65 years  + Essential services;  + Pregnant women, in the 2nd and 3rd trimester ;  + Individuals with morbid obesity;  + Individuals with asthma 6 months-65 years;  + Individuals with chronic respiratory disease, neuromuscular disease and immunosuppression from all ages. | 26/10/2009 |
|  | + Priority to children between 6 months – 2 years and individuals with chronic diseases 6 months – 65 years | 16/11/2009 |
|  | All children > 6 months – < 12 years. | 17/12/2009 |
| Romania | + Health care workers  + Essential services  + School children over 16 years  + Students  + Teachers | 26/11/2009 |
|  | All people aged over 16 years with priority to people with chronic diseases and pregnant women | 16/12/2010 |
| Spain | + People over six months of age belonging to high risk groups including obesity and pregnant women a any time of pregnancy  + Essential civil services  + Care givers of high risk persons | 16/11/010 |
