# Supplementary material for: Estimates of Pandemic Influenza Vaccine Effectiveness in Europe, 2009–2010: Results of Influenza Monitoring Vaccine Effectiveness in Europe (I-MOVE) Multicentre Case-Control Study
Source: PLoS Med. 2011 Jan 11;8(1):e1000388. doi: 10.1371/journal.pmed.1000388 (PMC3019108; doi:10.1371/journal.pmed.1000388)
Supplement: Table S3 — Estimated pandemic vaccination coverages by country study site, multicentre case-control study, influenza season 2009–2010, seven European Union study sites. (0.03 MB DOC) [file pmed.1000388.s005.doc]

Table S3: Estimated pandemic vaccination coverages by country study-site, multicentre case-control study, influenza season 2009-10, seven EU study sites

| Country | Estimated vaccination coverage | Source |
| --- | --- | --- |
| France |  | <http://www.afssaps.fr/var/afssaps_site/storage/original/application/e41f68fbee043b89e1fc740dac52d2e1.pdf> |
| Hungary | 26.8% of the total population | http://www.oek.hu/oek.web?to=839,1719&nid=41&pid=10&lang=hun File name: Honlapheti_2010_18 |
| Ireland | 23% of the population eligible for the vaccine | <http://ndsc.newsweaver.ie/epiinsight/viz7qx9a6lrqkeph6tk9uv> |
| Italy | 4.14% of the population eligible for the vaccine | <http://www.epicentro.iss.it/focus/h1n1/archivioflunews.asp> |
| Portugal | ~4.7% of the total population | *Ministério da Saúde. Comunicado da Ministra da Saúde a 04/02/2010 <http://www.portaldasaude.pt/portal/conteudos/a+saude+em+portugal/ministerio/comunicacao/comunicados+de+imprensa/ponto+gripe+vacinacao.htm> [accessed in 10/08/2010] |
| Romania | 8% of the population eligible for the vaccine | National Institute of Public Health, National Centre for Surveillance and control of Communicable Diseases - oral communication / presentation during the Regional meetings with epidemiologist anfd Familly doctors, 27, 28 May 2010 Bucharest, 4 June Timiosoara, Iasi, 11 June Baia Mare |
| Spain | 16.44% of the population eligible for the vaccine | Spanish Ministry of Health |
