# Supplementary material for: Estimates of Pandemic Influenza Vaccine Effectiveness in Europe, 2009–2010: Results of Influenza Monitoring Vaccine Effectiveness in Europe (I-MOVE) Multicentre Case-Control Study
Source: PLoS Med. 2011 Jan 11;8(1):e1000388. doi: 10.1371/journal.pmed.1000388 (PMC3019108; doi:10.1371/journal.pmed.1000388)
Supplement: Table S4 — Pooled crude and adjusted PIVE restricted to ILI patients swabbed <4 d after onset of ILI symptom, multicentre case-control study, influenza season 2009–2010, seven European Union study sites. (0.05 MB DOC) [file pmed.1000388.s006.doc]

**Table S4**

Pooled crude and adjusted pandemic vaccine effectiveness (PIVE) restricted to ILI patients swabbed < 4 days after onset of ILI symptom, multicentre case-control study, influenza season 2009-10, seven European Union study sites

|  |  | **Included population** | **N** | **PIVE %** | **95%  Confidence Interval** |
| --- | --- | --- | --- | --- | --- |
| **Complete case analysis** * | **Crude**‡ | All | 1369 | 77.2 | 52.0-89.2 |
|  |  | < 65 years | 1257 | 82.2 | 58.4-92.4 |
|  |  | 15-64 years | 827 | 74.9 | 40.3-89.4 |
|  |  | No chronic disease | 1087 | 79.2 | 46.9-91.8 |
|  | **Adjusted model ^∫^** | All | 1369 | 63.1 | 16.6-83.7 |
|  |  | < 65 years | 1257 | 69.5 | 23.9-87.7 |
|  |  | 15-64 years | 827 | 63.1 | 4.8-85.7 |
|  |  | No chronic disease | 1087 | 67.3 | 10.6-88.0 |
| **Imputed data ^†^** | **Crude‡** | All | 2728 | 81.3 | **65.7-89.8** |
|  |  | < 65 years | 2544 | 85.9 | 71.7-92.9 |
|  |  | 15-64 years | 1355 | 78.9 | 53.2-90.5 |
|  |  | < 15 years | 1182 | 93.8 | 73.7-98.5 |
|  |  | No chronic disease | 2224 | 83.1 | 64.3-92.0 |
|  | **Adjusted model ∫** | All | 2728 | 68.9 | 39.0-84.1 |
|  |  | < 65 years | 2544 | 76.5 | 49.8-89.0 |
|  |  | 15-64 years | 1355 | 70.8 | 29.4-87.9 |
|  |  | <15years | 1218 | 84.8 | 31.0-96.6 |
|  |  | No chronic disease | 2224 | 70.5 | 33.9-86.9 |

* Excluding individuals with missing values

† Missing data imputed using imputation using chained equations

‡ Study site included in the model as fixed effect

∫ Model adjusted for 2009-10 seasonal influenza vaccination, any influenza vaccination in previous two seasons, presence of at least one chronic disease, sex, at least one hospitalisation for chronic disease in the previous 12 months, current smoker, age group, practitioner visits in previous 12 months ( 0, 1-4 and 5+ visits), month of symptom onset (note: in the 15-64 years stratum no adjustment for age group; in the “no chronic disease” stratum no adjustment for chronic disease or hospitalisations for chronic disease
