# Supplementary material for: Estimates of Pandemic Influenza Vaccine Effectiveness in Europe, 2009–2010: Results of Influenza Monitoring Vaccine Effectiveness in Europe (I-MOVE) Multicentre Case-Control Study
Source: PLoS Med. 2011 Jan 11;8(1):e1000388. doi: 10.1371/journal.pmed.1000388 (PMC3019108; doi:10.1371/journal.pmed.1000388)
Supplement: Table S5 — Logistic regression model: Full model for complete case analysis. (0.11 MB DOC) [file pmed.1000388.s007.doc]

**Table S5**

## Logistic regression model

Full model for complete case analysis

|  | **N** | **Pandemic Vaccine Effectiveness** | **95% CI** |
| --- | --- | --- | --- |
| Crude | 1502 | 79.0 | 55.8-90.0 |
| Adjusted | 1502 | 66.0 | 23.9-84.8 |

| **Variable** | **Odds Ratio** | **95% Confidence interval** |  | **Likelihood ratio test** | **% difference** |
| --- | --- | --- | --- | --- | --- |
|  |  | **Lower limit** | **Upper limit** |  |  |
| Pandemic vaccination | 0.34 | 0.15 | 0.76 |  |  |
| Seasonal vaccination | 0.90 | 0.49 | 1.65 | 0.735 | 3.41 |
| Previous seasonal flu vaccination in past 2 yrs | 1.01 | 0.57 | 1.79 | 0.971 | 0.11 |
| Chronic disease | 0.96 | 0.64 | 1.46 | 0.865 | 0.36 |
| Sex | 0.94 | 0.72 | 1.21 | 0.614 | 0.16 |
| Hospitalisations for chronic disease in past 12 months | 0.41 | 0.11 | 1.50 | 0.145 | -1.59 |
| Current smoker | 0.68 | 0.43 | 1.09 | 0.102 | -2.17 |
| 15-64 years^1^ | 0.80 | 0.59 | 1.10 | 0.010 | -6.89 |
| 65+ years^1^ | 0.33 | 0.16 | 0.70 |  |  |
| 1-4 GP visits in past year^2^ | 0.86 | 0.59 | 1.25 | 0.000 | 2.54 |
| 5+ GP visits in past year^2^ | 0.47 | 0.31 | 0.70 |  |  |
| Onset in February^3^ | 0.52 | 0.28 | 0.99 | 0.000 | 31.58 |
| Onset in March^3^ | 0.33 | 0.04 | 2.60 |  |  |
| Onset in November^3^ | 7.53 | 4.78 | 11.87 |  |  |
| Onset in December^3^ | 5.46 | 3.67 | 8.13 |  |  |
| Country 2 | 1.56 | 1.02 | 2.39 | 0.003 | -29.19 |
| Country 3 | 2.48 | 1.43 | 4.30 |  |  |
| Country 4 | 1.01 | 0.55 | 1.89 |  |  |
| Country 5 | 0.73 | 0.34 | 1.61 |  |  |
| Country 6 | 0.99 | 0.54 | 1.81 |  |  |
| Country 7 | 2.45 | 0.73 | 8.28 |  |  |

^1^ Compared to 0-14 years of age

^2^ Compared to 0 GP visits in past year

^3^ Compared to onset in January

Full Model for imputed data

|  | N | Pandemic Vaccine Effectiveness | 95% CI |
| --- | --- | --- | --- |
| Crude | 2902 | 82.8 | 68.6- 90.6 |
| Adjusted | 2902 | 71.9 | 45.5-85.5 |

| **Variable** | **Odds Ratio** | **95% Confidence interval** |  | **Likelihood ratio test** | **% difference** |
| --- | --- | --- | --- | --- | --- |
|  |  | **Lower limit** | **Upper limit** |  |  |
| Pandemic vaccination | 0.28 | 0.145 | 0.544 | 0.000 | #N/A |
| Seasonal vaccination | 1.02 | 0.617 | 1.670 | 0.952 | -0.49 |
| Previous seasonal flu vaccination in past 2 yrs | 1.33 | 0.79 | 2.24 | 0.295 | 2.31 |
| Chronic disease | 1.12 | 0.79 | 1.59 | 0.538 | -1.26 |
| Sex | 0.87 | 0.73 | 1.04 | 0.139 | 0.88 |
| Hospitalisations for chronic disease in past 12 months | 0.51 | 0.20 | 1.35 | 0.180 | -1.57 |
| Current smoker | 0.63 | 0.41 | 0.96 | 0.034 | -2.00 |
| 15-64 years^1^ | 0.62 | 0.50 | 0.77 | 0.000 | -13.09 |
| 65+ years^1^ | 0.15 | 0.08 | 0.28 | 0.000 |  |
| 1-4 GP visits in past year^2^ | 0.86 | 0.64 | 1.17 | 0.353 | 5.34 |
| 5+ GP visits in past year^2^ | 0.41 | 0.29 | 0.59 | 0.000 |  |
| Onset in February^3^ | 0.41 | 0.24 | 0.71 | 0.002 | 36.84 |
| Onset in March^3^ | 0.25 | 0.03 | 1.93 | 0.186 |  |
| Onset in November^3^ | 7.37 | 5.44 | 10.00 | 0.000 |  |
| Onset in December^3^ | 5.45 | 4.08 | 7.28 | 0.000 |  |
| Country 2 | 1.55 | 1.10 | 2.19 | 0.016 | -28.52 |
| Country 3 | 3.19 | 1.96 | 5.21 | 0.000 |  |
| Country 4 | 1.13 | 0.63 | 2.02 | 0.690 |  |
| Country 5 | 0.75 | 0.37 | 1.54 | 0.436 |  |
| Country 6 | 1.16 | 0.68 | 1.98 | 0.579 |  |
| Country 7 | 2.94 | 0.98 | 8.82 | 0.057 |  |

^1^ Compared to 0-14 years of age

^2^ Compared to 0 GP visits in past year

^3^ Compared to onset in January
