# Supplementary material for: Estimates of Pandemic Influenza Vaccine Effectiveness in Europe, 2009–2010: Results of Influenza Monitoring Vaccine Effectiveness in Europe (I-MOVE) Multicentre Case-Control Study
Source: PLoS Med. 2011 Jan 11;8(1):e1000388. doi: 10.1371/journal.pmed.1000388 (PMC3019108; doi:10.1371/journal.pmed.1000388)
Supplement: Table S6 — Analysis using start of study period more than 30 d after the start of the study site-specific vaccination campaign. (0.04 MB DOC) [file pmed.1000388.s008.doc]

**Table S6**

### Analysis using start of study period more than 30 days after the start of the study site specific vaccination campaign

#### Complete case analysis

|  |  | Included population | N | PIVE % | 95%  Confidence Interval |
| --- | --- | --- | --- | --- | --- |
| **Complete case analysis^†^** | **Crude‡** | All | 1249 | 77.0 | 51.6-89.1 |
|  |  | < 65 years | 1130 | 81.8 | 57.4-92.2 |
|  |  | 15-64 years | 778 | 75.0 | 40.7-89.5 |
|  |  | No chronic disease | 983 | 80.3 | 49.8-92.2 |
|  | **Adjusted model 1^∫^** | All | 1249 | 62.1 | 14.6-83.2 |
|  |  | < 65 years | 1130 | 68.7 | 21.8-87.4 |
|  |  | 15-64 years | 778 | 61.0 | -0.9-84.9 |
|  |  | No chronic disease | 983 | 70.1 | 18.5-89.0 |

† excluding individuals with missing values

‡ Study site included in the model as fixed effect

∫ Model adjusted for 2009-10 seasonal influenza vaccination, any influenza vaccination in previous two seasons, presence of at least one chronic disease, sex, at least one hospitalisation for chronic disease in the previous 12 months, current smoker, age group, GP visits in previous 12 months ( 0, 1-4 and 5+ visits), month of symptom onset

NOTE: in the 15-64 years stratum we do not adjust further for age group. In the “no chronic disease” stratum we do not adjust for chronic disease or hospitalisations for chronic disease
