# Supplementary material for: Estimates of Pandemic Influenza Vaccine Effectiveness in Europe, 2009–2010: Results of Influenza Monitoring Vaccine Effectiveness in Europe (I-MOVE) Multicentre Case-Control Study
Source: PLoS Med. 2011 Jan 11;8(1):e1000388. doi: 10.1371/journal.pmed.1000388 (PMC3019108; doi:10.1371/journal.pmed.1000388)
Supplement: Table S7 — Number and proportion of observations (n = 2,902) with missing values by variable, influenza season 2009–2010, seven European Union study sites. (0.03 MB DOC) [file pmed.1000388.s009.doc]

**Table S7**

Number and proportion of observations (N=2902) with missing values by variable, influenza season 2009-10, seven European Union study sites

|  | Number of observations with missing values | Proportion of missing values (%) |
| --- | --- | --- |
| **Age** | 7 | 0.24 |
| **Sex** | 19 | 0.65 |
| **Fever** | 27 | 0.9 |
| **Headache** | 59 | 2.03 |
| **Cough** | 24 | 0.82 |
| **Sore throat** | 43 | 1.48 |
| **Days between onset of symptoms and swabbing** | 0 | 0.00 |
| **Diabetes** | 542 | 18.80 |
| **Heart disease** | 548 | 18.90 |
| **Any hospitalization in the previous 12 months for chronic diseases** | 578 | 19.90 |
| **Smoking history** | 349 | 12.03 |
| **Pandemic vaccination** | 67 | 2.31 |
| **Seasonal vaccination, 2009-10** | 14 | 0.48 |
| **Any influenza vaccination in the previous two seasons** | 1070 | 36.87 |
| **Median number of GP visits in the previous 12 months** | 799 | 27.53 |
